# Challenges of home care: a qualitative study

**DOI:** 10.1186/s12912-024-01878-0

**Published:** 2024-03-28

**Authors:** Mohsen Shahriari, Donya Hafezi Nia, Fatemeh kalij, Maryam Sadat Hashemi

**Affiliations:** 1 Nursing and Midwifery Care Research Center, Department of Adult Health Nursing, School of Nursing and midwifery, Isfahan University of Medical Sciences, Isfahan, Iran; 2 Nursing Student, Department of Psychiatric Nursing, School of Nursing and Midwifery, Donya Hafezi Nia, Isfahan University of Medical Sciences, Isfahan, MS Iran; 3 Department of Psychiatric Nursing, School of Nursing and Midwifery, Isfahan University of Medical Sciences, 4 Fatemeh kalij, MS, Nursing Student, Isfahan, Iran; 4 Nursing & Midwifery Care Research Center, Department of Nursing Critical Care, School of Nursing and Midwifery, Isfahan University of Medical Sciences, Isfahan, Iran

**Keywords:** Challenge, Home care, Nurses, Content analysis

## Abstract

**Background:**

Despite countless benefits of home care, unfortunately, the variety and quality of services provided by homecare centers are uncertain. This study was conducted to explore of home care challenges.

**Methods:**

The present qualitative study used the content analysis approach. A total of 17 participants, including nurses, managers of home care centers, and patients, were enrolled through purposive sampling. Data were collected using semi-structured interviews and analyzed through Granheim’s qualitative content analysis method.

**Results:**

In order to explain the challenges of home care, after analyzing the data, 700 primary codes, 15 initial categories, sub-subcategories and two main categories, including ‘infrastructural challenges’ and ‘challenges related to the process of home care services provision’ emerged. The main category, “infrastructural challenges”, consisted of 4 sub-categories (the challenge of acculturalization of home care services, economic challenges of providing services, challenges related to human resources, and the challenge of policymaking and setting regulations and rules for home care). The second main category, “challenges related to the process of home care services provision”, consisted of 2 subcategories: challenges of improving the quality of home care services and the challenge of facilities for service provision.

**Conclusion:**

In order to promote and improve the quality of home care services, in addition to providing insurance coverage for the services, acculturalization and revising the bylaws, empowering the human resource, enhancing the monitoring of the performance of home care centers, and employing modern technology need to be taken into account.

## Background

The demographic structure of Iran as a developing country has undergone various changes in recent years. As stated by the WHO, Iran is one of the three countries with the most dramatic population changes, and along with Chile and China, will have more older people than the United States in near future. The older population of Iran is expected to reach 25% by 2051 and makes this group the fastest-growing population in the country [1]. Fertility and mortality decline are major drivers of Iran’s population aging. A rapid and sharp fall in fertility rates over the past three decades as well as a substantial rise in life expectancy are causing rapid aging of Iran’s population. The speed of aging in Iran is truly remarkable, its population of 65 years and over will triple in 26 years (2023–2049) compared with 157 years in France, 100 years in the UK and 89 years in the US) [1]. Iran is ill-prepared for such a rapid population aging, it either has the economic foundation of these other countries nor the financial and institutional infrastructure that these countries developed over many decades. Providing care for a growing aging population will be one the most significant challenges faced by Iranian families [2].

The changes in the epidemiological pattern of diseases at old age and the prevalence and incidence of chronic diseases on the one hand, and the need of the healthy older people for healthcare on the other hand highlight the necessity of appropriate preventive and therapeutic interventions for the older people. Public health services and medical care are interrelated, and access to them along with some other factors lead to increased life expectancy worldwide.[4] When it comes to older people healthcare, the main goal is to maintain good health, so that these people can live an active life with their families in the environment in which they are, and maximize their physical and mental independence as well as social self-efficacy. Today, improved living conditions and increased life expectancy have led to the emergence of old age and the higher prevalence of chronic diseases in societies [3, 4]. Most patients with chronic diseases or those in the final stages of life receive the necessary services from hospitals. They often have prolonged and frequent hospitalizations, which increase the hospital’s bed occupancy rate and medical costs and reduce the efficiency of treatment centers. This is one of the most significant economic, social, and health challenges, particularly for healthcare providers [5]. In developed countries, home care is one of the pivots of the medical service program. Home care services have a significant role in preventing re-hospitalization, reducing hospital stay length and healthcare costs, and increasing the efficiency of healthcare centers. Continuing and following up on nursing care for clients after discharge from the hospital leads to mental peace for families and clients, reduces family costs, especially for those with a patient suffering from a chronic or incurable disease, and increases the participation in and empowerment of patients and their families in self-care [6–8]. Hashemzadeh et al.’s study (2023) showed that home care services after being discharged from the hospital play an important role in completing the provision of healthcare services. Home care services after being discharged from the hospital improves service delivery, reduces costs, and helps in achieving health goals. It also benefits patients, society and the government in various cultural and social fields [9]. According to the constitution of Iran, “Since the family is the basic pillar of the Islamic society”, all related laws, regulations, and programs should be aimed at facilitating the family formation and preserving its sanctity and the stability of family relationships.” Providing health care services at home can facilitate the implementation of this law by helping family members stay near their patients and care for the elderly at home. Accordingly, the preliminary bylaws for providing medical services at home and establishing nursing counseling centers were approved by the Ministry of Health and Medical Education in Iran in 1999, and these regulations were revised in 2014 [10]. The inclusive goal of establishing these centers was to directly and indirectly provide, maintain, and improve clients’/patients’ health by providing nursing care based on society’s care needs, consultation, education, treatment, rehabilitation, and supplying efficient human resources with emphasis on increasing health and reducing the impact of disability, especially in chronic and the elderly [11–13].

A study by Baraati et al. (2009) showed that home care reinforces attachment in the family, community, and health system, improves patients’ quality of life and the community’s health and reduces healthcare costs. Home care increases patient and family satisfaction (since provided in an intimate family environment), improves the quality of services and reduces medical errors and complications caused by hospital processes. Due to the above benefits, home care can play a major role in providing health services by taking advantage of the capacity of families, society, and NGOs and helping the development of community participation [14].

Numerous studies indicate that in countries where home care services are well-expanded, these services include a wide range of care, such as acute care, long-term supportive care, support for patients with mental disorders, rehabilitation, and end-of-life care [15]. This is while home care services are not specialized in Iran. Heydari et al.’s study (2016) showed that position for home care in the healthcare system, considering cultural dimensions in Iranian society and providing an appropriate infrastructure, can be beneficial to improve the situation of home care services in Iran. The data were divided into three main categories and eight subcategories. Main categories included treatment-based approach in the healthcare system (One-dimensional management in health system, priority of hospital services over community services and defect in the education system), cultural dimensions (community distrust to non-physician experts, defect in the safety of care providers and families), and the lack of adequate infrastructure (Lack of insurance coverage, absence of executive protocols and defect in the interdisciplinary cooperation) [11].Lotfi Fatemi et al. (2019) did a study to explore the challenges of delivering home care from the perspective of Iranian nurses. They categorized the various dimensions of home care challenges in five main categories including “difficult instances”, “economic problems”, “professional barriers”, “social difficulties”, and “bureaucratic tension”. In addition, the results of this study showed that the nurses in delivering home care experienced several economic, psychosocial, and bureaucratic problems. Facilitating the nursing processes, supporting home care, and recruiting nurses that had the potential to cope with the existing stressful factors and economic incentives can increase the quality of home care. The study was conducted from September 2016 to September 2017 in the provinces of Khorasan and Tehran in Iran [16]. Valizadeh et al. (2019) did an Integrative review to explore challenges and barriers faced by home care centers and their equivalents in Iran. The findings suggest that these problems could be classified in nine categories. They include the following: Non-application of standard and integrated methods for home care nursing services, deficiency in intra- and extra-organizational communications, absence of proper organizational infrastructure, lack of adequate and effective human resources, absence of legal and security supports, economic problems, information poverty, cultural constraints, and ignoring ethical issues [17]. Kianian et al.’s study (2022) showed that applying standard strategies to monitor the quality of services, paying attention to infrastructure, and having a clear framework for policy-making are beneficial for the development of the home health care in Tabriz, Iran [18].

There are few studies conducted to inspect the challenges faced by home care nurses in Iran; therefore, there is demand for further studies in this sector of Iran’s healthcare system. On the other hand, home care nursing challenges in Iranian society was influenced by cultural and social factors.The home care nurse usually has a close relationship with family members and the patient, so it seems that he/she has a close relationship with the social structure and family customs and has his/her own problems.In fact, nurses’ perceptions of the crisis are shaped by the complexities of laws, patients, families, and home care agencies. To further explore these aspects, interviewing health care personnel could be a method to achieve more precise insight and understanding this situation. Currently, there are many challenges in providing home care services in the city of Isfahan. Qualitative studies that have been conducted in Iran in the field of home care challenges were carried out in the cities of Tehran, Tabriz and Khorasan. Considering the differences cultural and social differences among the people of different cities of Iran, so the results of these studies cannot be generalized to the city of Isfahan. Therefore, this study was conducted to investigate the challenges of home care in Isfahan city.

## Methods

This is a qualitative content analysis conducted on Challenges of Home Care in 2022.

### Participants and sampling method

A purposeful sample of 17 homecare staff recruited from a convenience sample of ten municipalities in Isfahan city was included. Data collection continued until data saturation, when no new data was obtained from the interviews. A purposive sample was employed to recruit registered nurses (RN) and managers of home care centers with the inclusion criteria. The inclusion criteria included nurses and managers of home care centers with at least two years of work experience in home care centers and no hearing, speech, or known mental disorders The inclusion criterion for the participants was that they were members of the staff in homecare services; their selection was based on their potential to contribute important and balanced information to the study. The participants included both leaders and staff of various ages, gender, educational levels, socio-economic level, shift rotation, and type of home care center and years of experience. Permission to recruit participants from the home care team was obtained by the senior manager. The exclusion criteria included unwillingness to continue cooperating in the study. Three participants withdrew from the study and, hence, 17 interviews were included in the final analysis. Some people rejected their participation offer stating that they did not have spare time for this research, whereas others claimed that they did not want to participate or that they did not have anything to contribute.

### Data collection

Each interview lasted approximately 45–60 min.

The data collection was performed using semi-structured individual interviews from October 23, 2021, to July 21, 2022 The interview location included the offices of home care centers, deputy of treatment, nurses’ resting rooms in the general and critical wards of teaching hospitals, nursing faculty rooms, or any other place suggested by the participant.

We used a qualitative survey asking open-ended questions to allow space for longer narratives and information from respondent [19].Utilizing open-ended questions enables researchers to collect holistic and comprehensive information on the studied issues [20].Before the formal interview, we pre-interviewed a nurse and adjusted the interview questions according to the interview outcomes. The final interview questions included in the interview guides are presented in Table 1. The interviews continued until the data saturation and repetition of statements. No new codes were obtained after the eighth interview. These interviews were recorded using a mini-recording device and lasted from 45 to 60 min. Field notes were taken during and after interviews. After stating the study objectives and obtaining participants’ permission, the interviews were recorded using a digital recorder, transcribed verbatim using Word 2013, and analyzed.

**Table 1:** Open-ended questions in the qualitative survey

• What kind of home care services do people need and pay attention to? • What kind of home care services are most needed and noticed by people? • What is your opinion about the quality of providing home care? • In your opinion, what are the obstacles (cultural, legal, financial, manpower, equipment, and technology) to improving the quality of home care?
• According to the current conditions and facilities, what solutions do you suggest to improve the quality of home care?

### Data analysis

Data analysis was performed simultaneously with data collection using the qualitative content analysis approach proposed by Graneheim and Lundman. Recorded interviews were transcribed word for word; then, transcripts were perused and meaning units were identified. The researcher uses the names and categories derived from the data and obtain a novel insight through prolonged involvement and immersion in data [21].Then, expressions or words related to the concept under study were determined, primary codes were generated, conceptually similar codes were placed in a cluster, semantically related clusters were placed in a category, and finally, similar categories were merged.

The encoding and the initial development of the categories were mainly performed by the first and corresponding Author. All the interviews were transcribed verbatim. The transcribed interviews were first separately reviewed by both MH and MSH to gain an understanding of their content, and MH offered MSH explanations on them if necessary MH and MSH then separately determined the meaning units and condensed meaning units of three select interviews. In the next step, the researchers discussed the agreed-upon meaning units and the condensed meaning units. The condensation of the meaning units of the remaining transcribed interviews was performed by MH and discussed with MSH. Codes were assigned to the condensed meaning units by MSH and MH. Finally, six subcategories and two categories emerged after MH compared the codes in terms of their similarities and differences. The categories and subcategories were discussed again with MSH. It is worth noting that the codes determined by MSH and MH did not differ significantly from each other.

### Rigor

Trustworthiness was ensured using the confirmability, credibility, dependability, and transferability criteria [22]. Credibility was ensured through attended the research setting for about ten months to collect data and made an effort to collect the data by prolonged involvement in conducting interviews. In this study, in-depth interviews were used to obtain deep data, and participants were selected with maximum diversity (in terms of age, gender, education level, socio-economic level, work experience, shift rotation, and type of home care center). Moreover, the researcher used the member-check method to verify the accuracy of the extracted data and codes or to modify them. After coding five interviews, their transcription, along with the assigned codes, were returned to the participants to ensure the accuracy of the codes, and in case the codes did not match their point of view, they were modified. The peer review method was also used to verify the credibility of the data. To this end, three knowledgeable individuals who were experienced in qualitative research and were not involved in this study reviewed the data, the coding stages, and the emergence of categories to ensure the adaptability of codes and categories with the data.

In order to data transferability, the researcher made an effort to apparently present the study stages. Therefore, other researchers could follow the research process and judge the data transferability. Dependability was ensured through the researcher tried to document and record the events and decisions made during various stages of the study, including interviews and data analysis, in detail to provide the auditing opportunity for others. Confirmability was maintained by the researcher attempted to identify his attitude toward home care challenges and avoid bias in all stages of the study. Besides, he provided some parts of the interviews and their transcripts, along with the assigned codes, to three colleagues who were experts in qualitative research to confirm coding accuracy [23].

## Results

In order to explain the challenges of home care, 17 individuals were interviewed, of whom 9 were nurses, 6 were home care center managers, and 2 were family members of patients who had received home care services (Table 2). After analyzing the data, 700 primary codes, 15 initial categories, six subcategories, and two main categories, including ‘infrastructural challenges’ and ‘challenges related to the process of providing home care services,’ emerged, which are shown in Table 3.

**Table 2:** Characteristics of study participants

Participants	Age	Gender (Male/Female)	Education	Average Work Experience (Years)	Job
P1	40	Female	Master’s Degree	18	Supervising The Education Of The Patients Of Isfahan’s Vice-Chancellor Of Treatment
P2	63	Male	Master’s Degree	35	Director of Home Care Center and Director of Charity Institute
P3	54	Male	bachelors	32	Home Care Center Manager
P4	45	Female	bachelors	17	Home Care Center Manager
P5	35	Male	bachelors	17	Home Care Center Manager
P6	35	Male	bachelors	13	Home Care Center Manager
P7	42	Male	bachelors	20	Nurse
P8	28	Male	bachelors	7	Nurse
P9	32	Male	bachelors	9	Nurse
P10	37	Male	bachelors	12	Nurse
P11	27	Male	bachelors	5	Nurse
P12	52	Female	bachelors	30	Nurse
P13	22	Female	bachelors	1	Nurse
P14	51	Female	Master’s Degree	28	Nurse
P15	27	Male	bachelors	5	Nurse
P16	48	Male	bachelors	-----	patient
P17	58	Male	bachelors	-------	Patient

**Table 3:** Subcategories, Sub-Subcategories, and Main Categories

Main category	Subcategory	Sub-subcategory
Infrastructure challenges	The challenge of acculturalization of home care services	The challenge of informing
		Challenges of patient referral
	Economic challenges of providing service	The challenges of providing and allocating the required resources
		Challenges related to insurance
		Challenges of service pricing
	Challenges related to human resources	The challenge of recruiting, supplying, and adjusting human resources
		The challenge of workforce training
		The challenge of maintaining human resources
	The challenge of policymaking and setting regulations and rules for home care	The challenge of securing human resources
		The challenge of obtaining a license
		The challenge of revising regulations
challenges related to the process of home care services provision	Challenges of improving the quality of home care services	The challenge of empowering human resources
		The challenge of monitoring the performance of home care centers
	The challenge of facilities for service provision	The challenge of providing medical equipment
		The challenge of ignoring technology in providing services

### Infrastructure challenges

The main category, ‘infrastructural challenges,’ consisted of four subcategories (the challenge of acculturalization of home care services, the economic challenges of providing services, challenges related to human resources, and the challenge of policymaking and setting regulations and rules for home care).

### The challenge of acculturalization of home care services

This sub-subcategory was formed from two subcategories: the challenge of informing and the challenges of referring patients.

#### The challenge of informing

The participants of this study pointed out that one of the reasons for overlooking home care services was their lack of awareness of the relevant services. They believed that the media and the health education offices in hospitals should have been used for introducing home care centers and providing information about the type of home care services and approved tariffs. In addition, in order to register the home care centers’ telephone numbers in the city directory inquiries, the necessary correspondence should be done. One of the home care center managers stated:

Informing people through public media is necessary to make them aware of the rules and tariffs so that if a person visits them at home illegally, charges an inappropriate tariff, or provides inappropriate services, they can complain and follow up. We know these issues, but those related to financial and human resources and insufficient infrastructure in the country are among the obstacles and challenges. We have tried to improve home care. First, we integrated counseling centers and notified all hospitals. We also visited the centers and corresponded with the city directory inquiries so that these centers’ contact numbers are available and registered.”(p3).

### challenges of referring patients

Participants’ experiences showed that the provision of services was not continuous, and the majority of patients were unable to care for themselves after being discharged from the hospital; however, they were not referred to home care centers by doctors or the health education offices in medical centers. In order to improve the referral status, doctors should be aware of the list of home care centers available in the deputy of treatment and refer the patient to them. In this regard, the necessary cultural promotion should be considered. One of the managers of home care centers said:

*Another obstacle is the non-referral of the patient by the hospital, doctors, or health centers. When a patient is discharged, nobody tells them that if they need care, they can use such centers. They don’t provide them with the centers’ phone numbers. Doctors work with certain personnel and don’t refer patients to centers.* (p2).

### Economic challenges of providing home care services

This subcategory includes challenges in supplying and allocating required resources, insurance-related challenges, and challenges of service tariffing.

#### Challenges of supplying and allocating required resources

The participants pointed out that due to the lack of insurance coverage for the services, the establishment of home care centers is not cost-effective, and considering the current economic conditions, the majority of individuals cannot afford to pay the costs. In order to introduce the home care center and increase the number of customers, they provide services for a lower price than the approved one and sometimes with no charge and rely on the charities to provide resources. The managers of the home care centers believed that in order to attract people, the costs of home care should be adjusted through cooperating with other organizations such as insurance, Red Crescent, welfare and international organizations, attracting individuals’ and NGOs’ contributions, identifying donors and negotiating with them for financing, and managing and monitoring the financial resources donated by benefactors and individuals.

One of the nurses said:

*“We faced the problem of caring for end-of-life patients, those who were no longer provided with services by the hospital and, most importantly, they couldn’t afford to cover home care costs. Then, we were able to win the support of the benefactors of Isfahan by winning the trust of the citizens.”*(p11).

#### Challenges of service tariffing

Participants’ experiences showed that considering the cost of commuting, the cost of medical equipment, and the identical tariffs during holidays, working days, or at different times of day and night, providing home care services with approved tariffs were not affordable for them.

One of the managers of home care centers stated:

*Another challenge is the notified tariffs for home care, which is identified by the Cabinet of Ministers every year. For example, this year (2021), the tariff specified for IV injection isn’t compatible with the current economic conditions, and counseling centers complain about it. A set of specified tariffs similarly don’t match the society’s existing conditions. The center decides to send a nurse for home care; for services such as catheter insertion, the equipment must be taken too, and the cost charged is not a reasonable tariff and does not match the society’s conditions*(p4).

#### Insurance-related challenges

The participants mention that one of the issues of providing home care services is economic challenges. Due to notified high tariffs and lack of insurance coverage, most individuals cannot afford to pay for home care. Insurances are required to cover the relevant services to encourage individuals to use these services. Cooperating with other organizations such as insurance, Red Crescent, welfare, and international organizations, attracting people’s and NGOs’ participation in all ways possible to provide resources, leads to better acceptance of home care services.

One of the managers of home care centers said: “*According to the announcement of insurance organizations and the Ministry of Health, providing home care services has many benefits such as reducing the patient cost and insurance companies’ out-of-pocket payments, preventing hospital beds occupancy, and reducing the possibility of a patient contracting a hospital infection. Despite being acknowledged, the insurance laws of these issues are being followed slowly in the Supreme Council of Health Insurance and have only been implemented as a pilot study for some diseases in several provinces but have not yet in the whole country*.”(p2).

### Challenges related to human resources

The subcategory of human resources challenges was formed from the sub-subcategories: the challenge of attracting, supplying, and adjusting human resources, the challenge of training human resources, the challenge of preserving and retaining human resources, and ensuring the safety of human resources. From the perspective of some participants, home care centers’ employee screening and selection system should be revised. Home care centers should improve home care service quality by employing authorized, expert, and efficient staff, cooperating with experienced nurses and using interviews to select staff. Ensuring nurses’ safety was another challenge in providing home care from the nurses’ point of view. The participants stated that sometimes they experienced challenging interactions such as verbal violence, sexual harassment, and stigmatization of female employees, and the police should be aware of the nurse’s presence at home.

One of the participants said:

;*The more our services become scientific, experienced nurses and those holding a master’s degree are invited to cooperate, and non-professionals are prevented from entering this field, the better the quality of our services will be, which is for the benefit of the patient*.”(p10).

### The challenge of policymaking and setting regulations and rules for home care

This subcategory was derived from two sub-subcategories: ‘the challenge of obtaining a license’ and ‘the challenge of revising regulations.’

#### The challenge of obtaining a license

Participants’ experiences showed that the high rate of bureaucracy and prolonged processes for obtaining a license for establishing home care centers as an obstacle to developing home care services. Home care center managers stated that an extended and arduous administrative process must be completed to register a nursing and medical service company and obtain a license.

One of the participants said: “*The company registration process takes so much time that before you register the company and implement the idea, competitors implement the same idea; it is a waste of time. They also have cumbersome regulations, such as having an office, while our goal is to work on an online platform.”*(p12).

#### The challenge of revising regulations

The participants believed that the revision of the bylaws on the performance of home care centers, supervision of the implementation and compliance with laws and regulations, the revision of home care policies by decision-making groups, the compilation of bylaws for the specialization of home care centers, and the integrity of uniforms, records, and identification cards or labels should be taken into account. Moreover, regulations on the establishment of hospices should be approved. Today, the number of patients with hard-to-treat diseases has increased, and most of these patients need places to provide high-quality spiritual, psychological, social, and physical services to end-of-life patients. There should be regulations on establishing these centers, patient admission and discharge, coordination to continue patient treatments, and provision of financial resources for these centers.

One of the participants stated:

*“The support of liability insurance is poor. If our patient is injured or dies during the operation, we aren’t supported by the insurance, nor legally. Liability insurance must be defined accurately, so we can receive more legal support. There are strict rules for the center, from its area to the existence of a technical assistant. We can’t provide some services in the center and have problems in contracting.”*(p14).

### Challenges related to the process of home care services provision

This main category emerged from two subcategories: ‘challenges of improving the quality of home care services’ and ‘the challenge of providing facilities.’

### The challenges of improving the quality of home care services

This subcategory was derived from two sub-subcategories: the challenge of empowering human resources and the challenge of monitoring the performance of home care centers. Participants believed that in order to improve the quality of services, the processes of governance, policymaking, supervision, providing and empowering human resources, and improving the quality of the staff screening and recruitment system should be taken into account.

#### The challenge of empowering human resources

Due to the fact that home care was not included in the curriculum of undergraduate nursing students and nursing literature, and there was a lack of specialized training in the field of home care, this concept was incomprehensible to nurses, and they lacked a precise perception of it and its significance.

One of the participants said:

*a credit or webinar under the title of home care can be very helpful. In addition, webinars and regular training workshops should be held on patient care, the proper performance of procedures, and patient training.*(p1).

#### The challenge of monitoring the performance of home care centers

Among other challenges of providing home care from the nurses’ point of view were the challenges related to monitoring the provision of services. According to the participants, maintaining the quality and standards of care was necessary to improve the quality. Moreover, the participants pointed out the significance of monitoring and validation. They believed that succeeding in providing optimal home care services is determined by the practical assessment of the services. They stated that the presence of non-professionals, the provision of services by unauthorized centers and service companies, and not recruiting experts affected the quality of service provision, and the inspectors of the deputy of treatment should prevent moral and legal violations through periodic visits to the home care centers. They also believed the monitoring team could communicate with the family who received nursing services and evaluate their satisfaction with the provided services over the phone. Furthermore, the participants pointed out that professionals were not employed in most home care centers, affecting service quality and patient safety. Therefore, the authorized centers should have more supervision of care provision by home care centers. On the other hand, some centers charge patients regardless of the approved tariffs, which leads to dissatisfaction with the services provided.

One of the participants said:

*“One of our main challenges is the presence of unprofessional and unauthorized individuals in the field of home care and service provision. The regulations say only those who are either the founder of a nursing consultation center or have a contract with one of the nursing consultation centers can enter the home. But in our country, home care is provided in an unauthorized and illegal way. Sometimes, practical nurse assistants or service personnel learn something in hospitals and perform them later as home care.”* (p1).

### The challenge of facilities for service provision

This subcategory was extracted from two sub-subcategories: the challenge of providing medical equipment and the challenge of ignoring technology in providing services.

#### The challenge of providing medical equipment

Another challenge of providing home care from the perspective of nurses and managers of home care centers was the provision of medical equipment. The participants said that in order to alleviate this problem, home care centers should have had a contract with medical equipment centers, and these centers should have been granted bank loans to supply equipment.

One of the participants said:

*“We don’t have access to serum and medicines and have to prepare our equipment personally, which requires a lot of investment; we don’t have an electroshock device, so we can’t provide all services at home; for patients with unstable conditions, we call the emergency.”*(p10)

#### The challenge of ignoring technology in providing services

The participants pointed to the absence of technology as one of the obstacles to providing home care. They believed that telemedicine technologies, including telemedicine services, telenursing, and effective use of electronic medical records, had not been developed. The hospitals’ electronic systems should be designed with the ability to send history reports, disease diagnoses, case summaries, treatment measures, and consumed drugs to home care centers. Participants also pointed out the necessity of designing an integrated patient care system and application.

One of the participants said:

*“We should have access to the doctor’s order online because paper prescriptions aren’t available now. We should be able to check the prescription and the doctor’s order because if the patient asks us to inject an ampoule, we should be able to check the prescription. An application must be designed so we can all be linked together. This application will also make the deputy of treatment monitor our performance. It must have GPS, and when someone calls, they send the nearest center, like the central operator of the emergency, to provide services. There should be a possibility to consult online with specialist doctors at home. Sometimes we visit the patient and recognize that the patient needs a doctor or we need a consultation. It can be perfect if we communicate with the doctor immediately, especially through a video call. Like a counseling session, we explain the patient’s condition, take their recommendations, and provide the service.”(p7).*

## Discussion

In the present study, the participants considered the infrastructural challenges such as challenge of acculturalization of home care services, economic challenges of providing services, challenges related to human resources, and the challenge of policymaking and setting regulations and rules for home care, as one of the most significant obstacles to quality home care services. The results of Shahsawari et al.’s study (2018) likewise point to the fact that policy-making challenges (multidimensional management in the structure of the healthcare system, nurses as the pivot of the home care team, and community-based healthcare system) and the lack of necessary infrastructure (readiness of the infrastructure of the health system, social understanding of home care, social understanding of nurses, insurance coverage of service, appropriate electronic communication, sufficient knowledge about home care, and training professionals) affect the quality of home care services [24]. Heidari et al.’s study (2016) similarly indicated that the treatment-based approach in the healthcare system (treatment-oriented approach in the health system, one-dimensional management in the healthcare system, priority of hospital services over social services, and deficiencies in the educational system), cultural barriers (society’s distrust in non-physicians and deficiencies in care providers’ and families’ safety)’ and lack of sufficient infrastructure (absence of insurance coverage, lack of administrative protocols, and deficiencies in interdisciplinary cooperation) impact the quality of home care delivery [11]. The results of Moradian et al.’s study (2016) suggested that policy-based, family-based, and agency-based barriers were among the most significant barriers to providing home care. The leading policy-based barriers included legal and insurance restrictions and an ineffective network for social support. The absence of a professional team, the lack of an independent home care organization, and problems related to physicians’ visits were agency-based obstacles. In addition, the family-oriented obstacles included the family’s fatigue, anxiety, and the financial inability to pay the expenses [25].

One of the infrastructures requiring improvement for the development of home care services is financial problems. Findings show that home care services are not cost-effective for professionals, families, and agencies since they are not covered by insurance organizations. As far as economic aspects are concerned, nurses believe that the costs of home care are high and the caregivers’ income cannot compensate for these costs. Many home care agencies in the UK and US have reportedly gone bankrupt [26]. Other studies have shown that the financial crisis has impacted home care services and led to some disruptions in care delivery. A significant economic problem, It is that home care services in Iran are not covered by health insurance. Home care services in Iran face development obstacles due to lack of insurance coverage, with hospital services insured but community services uninsured. The healthcare system is a public-private partnership, with funding from government, insurance organizations, and patient co-payments. Currently, the health system funding in Iran comes from the government (23.8%), health insurance (30.6%), out-of-pocket payments (35.2%), private health insurance (6.1%), and individual donations and other sources (4.3%), based on data for 2018. Multiple insurance funds, uncoordinated decision-making, and inefficient financing schemes are the main challenges for Iran’s health insurance industry [27]. This problem results in limited access to home care services. Instead, families receive help from ineligible individuals who provide services at a lower cost. This leads to an inability to control the quality of home care. Insurance coverage therefore represents an effective factor in maintaining the quality of home care [28, 29]. Strong financial performance is associated with improved patient reported experience of care, the strongest component distinguishing quality and safety. The lack of set tariffs represents a further challenge for nursing staff and leads to situations of unequal and unfair competition. This led to the legal gap, i.e. unauthorized individuals can easily have access to a home care market, while licensed home care staff have many expenses in the same market. For this reason, the motivation of nurses to enter the market is reduced. Therefore, the role of macro planning in health and health policy is considerable to manage the pressures. Realistic targeting, well planning, and well organizing could make the current system more cost-effective, equitable, and accessible to all of the population [16].

In line with the results of the present study, other studies have emphasized that individuals are impelled to use low-cost, non-standard, and low-quality services due to high-cost official home care services. As a result of financial problems, most of these centers employ less knowledgeable and less skillful workers to provide more cost-effective services, and this defective cycle is reinforced. In addition, the majority of these centers lack the necessary licenses, and there is no comprehensive list of all legal and illegal medical centers in Iran. Therefore, insurance organizations’ support for home care institutions can effectively prevent challenges and development of home care services in the community [10, 18].

One of the infrastructural issues that must be considered necessary for developing home care services is educational gaps. Other studies have also indicated that home care services have been overlooked in academic and public health education programs. Professional education is primarily focused on preparing professionals for hospital settings. Shahsavari et al. (2018) and Kianian et al. (2022) likewise pointed to educational barriers as one of the barriers to providing quality home care services [18, 24].Training is required to prepare the healthcare system and society to provide and receive home care services. Numerous studies have accentuated the need for specialized training and the expansion of evidence-based certificates to support this valuable care. It is recommended that university educators collaborate with researchers to integrate evidence-based guidelines and develop programs to increase public knowledge [18].

Another challenge for providing home care from nurses’ point of view was the acculturalization of home care services. The study by Lotfi Fatemi et al. (2018) showed that nursing professionalism at home was influenced by various confounding and facilitating factors. Individual and professional skills, such as theoretical knowledge and practical skills, work experience, situation management, communication skills, ethical commitment, and professional, individual, and social values, were among the facilitators. However, weak management and training systems, society’s negative attitude toward home care nurses, and social deficiencies were identified as obstacles to professionalization in home care. Regarding compliance with the law and responsibility, home care nurses are responsible for their actions toward patients [30].

From the nurses’ perspective, ensuring nurses’ safety was another challenge in providing home care. Heydari et al. (2016) pointed to defects in care providers’ and families’ safety as one of the obstacles to providing home care [11]. The study by Grasmo et al. (2021) showed that challenging interactions, such as clients’ sickness or death, verbal violence, sexual harassment and stigmatization of female employees, unpredictable working conditions, and health-threatening risks (difficult driving and traveling conditions, snowy weather in winter, slippery roads, skidding and falling hazards, unhealthy air and unpleasant odors, dust and dirt, clutter, garbage, tobacco smoke, and exposure to cigarette smoke) reduces nurses’ safety during home care. The results of the study by Grasmo et al. indicate that in some areas, the rate of crime, drug use, and violence is high, affecting nurses’ safety during home visits [31]. Arab et al.’s study (2022) similarly pointed out the significance of nurses’ safety and privacy in providing home care. These researchers stated that observing communication boundaries in patient care improves nurses’ safety [32].

Another significant infrastructural obstacle is legal gaps. The findings showed that the existing security, advertising, and safety laws need to be more comprehensive and transparent. According to the results of the present study, Fatemi et al., (201 9) state that the home care regulations need to be more apparent and comprehensive. The interpretation of the law varies in each ward, and each department bears its own opinion [16]. Rosenfeld (2021) considered it essential to revise the laws and legal mechanisms of home care [33]. However, other studies showed that home care regulations are required to be transparent, accessible, and comprehensive since a deficit in the home care regulations is detrimental to caregivers and their clients [34, 35]. Lack of standards, apparent and specific laws, regulations, and instructions about what and how home managers and nurses care should do, lead to insufficient supervision, thus reducing the quality of home care services.

Another challenge was related to the unfavorable quality of the process (lack of staff training to provide home care, non-standard health assessment before performing procedures, non-standard patient education, and non-standard home care). In the present study, a deficit in accreditation as a tool was identified as another perceived barrier. Accreditation is a tool to evaluate and guarantee the quality of services, which was not sufficiently investigated in previous studies. In a study, Cristofori et al. (2020) stated that the accreditation of home care services is often performed using non-specific tools that are unable to accurately assess the quality of services [36]. According to the National Institute of Home Care Accreditation, it is essential to establish fundamental national standards for proper home care practices. In addition, the home care providers’ competencies should be continuously evaluated using an independent accreditation process, and staff needs to receive ongoing educational programs based on accreditation standards. The results of the study by Kianian et al. (2022) likewise refer to inappropriate accreditation as one of the obstacles to providing quality home care services [18].The results of the study by Tang et al. (2020) likewise indicate that in order to improve the quality of care, there should be apparent rules on how to provide home care [37]. So far, no specific organization has been in charge of inspecting and evaluating home health centers. After establishing the nursing department in the Iranian Ministry of Health, Medicine, and Medical Sciences, this department has supervised these centers and seeks to identify legal and competent centers and find a system to follow up on patients’ complaints. Universities of medical sciences still lack accurate statistics on such centers in their region or province [10]. Tang et al.’s study (2020) showed that the infrastructure, processes, and evaluation system of home care services in Shanghai, China, were not satisfactory, and strategies such as issuing standards to evaluate the quality of home care, modification of the quality of the structure and the process of providing home care services, and the creation of a service quality evaluation system were necessary to improve the quality of home care services in China [37].

According to nurses, the lack of medical equipment was another challenge in providing home care. The results of Tang et al.’s study (2020) similarly indicate that insufficient medical resources and equipment and the lack of human resources and vehicles for home visits affect the quality of service delivery. Another challenge of providing home care from the nurses’ point of view was inadequate human resources. Staffing is a critical prerequisite for ensuring quality home care. The participants acknowledged a significant lack of human resources to provide home care services, which led to limited and undeveloped home care [37].

In the present study, the participants pointed to the absence of technology as one of the obstacles to providing home care. Ajami et al.’s study (2018) indicate that despite the significant, up-to-date, and fast-paced developments in employing information and communication technology at the world level, Iran suffers from two fundamental challenges. First, home care services in Iran are in the infancy of exploiting information and communication technologies. Secondly, home care center managers and investors lack sufficient knowledge of information and communication technologies, or their knowledge is not outdated. In Iran, remote care systems and technologies, including remote health services, nursing, consultation, evaluation, and effective use of electronic medical records, have not been developed. Considering the novelty of using information and communication technologies and home care, Iran is at the beginning of the path compared to other developing countries. However, the necessity of using information and communication technologies is highlighted considering the increasing trend of population aging, high requests to receive home care services, and the variety of services [38] Technology has the potential to minimize communication errors in home health care. For instance, electronic medical records can improve communication and reduce transition and handoff errors.It is important to note that home care may be less susceptible to some of these errors than hospital care. For example, in the home setting, providers tend to have a longer relationship with the patient, staff relationships tend to be personal rather than team-based, and there may be fewer interruptions in treatment. Alternatively, certain types of communication errors may be more likely in home care, especially when multiple conflicting family members are involved and there is no formal communication and transition system. The results of Jones et al.’s study (2017) suggest that in order to improve the quality of home care services, home care nurses should have advanced access to hospital records and direct phone lines to communicate with hospital nurses to resolve ambiguities, assess needs, and determine care goals by meeting patients in the hospital. For drug management, negotiators of care agencies should communicate directly with physicians or pharmacists to resolve disputes [39].

### Practical and political implications

According to the results, there are numerous challenges and barriers to providing home care services; these are often intertwined so that each leads to another. This wide range of obstacles requires coordination and a precise and comprehensive planning by the government, academic experts, and nurses so that they can provide an effective model for assessing, planning, implementing, and evaluating home care in Iran.

### Limitation

The limitation of the present study was that some participants refused to give complete information or self-censored, which was solved to a large extent by assuring the confidentiality of the interviews and asking probing questions. Also this study has been conducted by qualitative method; therefore, the findings of this study do not have the ability to be generalized to other communities.

## Conclusion

There are many structural and process challenges in providing home care services in Iran. By considering these challenges more meticulously, it seems that solving the infrastructural problems and challenges of this domain can extensively prevent the genesis of challenges tied to other functions due to the strong influence of this domain on others. The utilization and acceptance of these services requires culturalization and investment of sufficient time and resources and insurance. For improving quality of home care, training specialized human resources in the home care, monitoring and accreditation are other issues that need to be considered.

## Data Availability

Not applicable.

## References

[1] Mehri N, Messkoub M, Kunkel S (2020). Trends, determinants and the implications of population aging in Iran. Ageing Int.

[2] Bahmaei J, Ravangard R, Bahrami MA, Asadollahi A, Bastani P (2023). Policy requirements in promoting older people health care in Iran: a qualitative study. J Educ Health Promotion.

[3] Brown MM. Transitions of care. Chronic illness care: Principles and practice. 2018:369– 73.

[4] Rodrigues RAP, Bueno AA, Casemiro FG, Cunha ANd C, LPNd, Almeida VC (2019). Assumptions of good practices in home care for the elderly: a systematic review. Revista Brasileira De Enfermagem.

[5] Dale MC, Helton MR. Nursing home care. Chronic illness care: Principles and practice. 2018:245– 57.

[6] Arsenault-Lapierre G, Henein M, Gaid D, Le Berre M, Gore G, Vedel I (2021). Hospital-at-home interventions vs in-hospital stay for patients with chronic disease who present to the emergency department: a systematic review and meta-analysis. JAMA Netw open.

[7] Roberts B, Robertson M, Ojukwu EI, Wu DS. Home based palliative care: known benefits and future directions. Curr Geriatr Rep. 2021:1–7. 10.1007/s13670-021-00372-8 PMC8614075 34849331

[8] Glasdam S, Ekstrand F, Rosberg M, van der Schaaf A-M (2020). A gap between the philosophy and the practice of palliative healthcare: sociological perspectives on the practice of nurses in specialised palliative homecare. Med Health Care Philos.

[9] Hashemzadeh Z, Habibi F, Dargahi H, Arab M (2023). Explanation of the benefits and challenges of Home Care Plan after Hospital Discharge: a qualitative study. Payavard Salamat.

[10] Nikbakht-Nasrabadi A, Shabany-Hamedan M (2016). Providing healthcare services at home-a necessity in Iran: a narrative review article. Iran J Public Health.

[11] Heydari H, Shahsavari H, Hazini A, Nasrabadi AN. Exploring the barriers of home care services in Iran: A qualitative study. Scientifica. 2016;2016. 10.1155/2016/2056470 PMC4835654 27127677

[12] Plöthner M, Schmidt K, De Jong L, Zeidler J, Damm K (2019). Needs and preferences of informal caregivers regarding outpatient care for the elderly: a systematic literature review. BMC Geriatr.

[13] Kinney J, Haapala D, Booth C. Keeping families together: the Homebuilders model. Routledge; 2017.

[14] Barati A, Janati A, Tourani S, Khalesi N, Gholizadeh M (2010). Iranian professional’s perception about advantages of developing home health care system in Iran. Hakim Res J.

[15] Walker CC, Druckman A, Jackson T. A critique of the marketisation of long-term residential and nursing home care. Lancet Healthy Longev. 2022. 10.1016/S2666-7568(22)00040-X 36098302

[16] Fatemi NL, Moonaghi HK, Heydari A (2019). Perceived challenges faced by nurses in home health care setting: a qualitative study. Int J Community Based Nurs Midwifery.

[17] Valizadeh L, Zamanzadeh V, Saber S, Kianian T. Challenges and barriers faced by home care centers: an integrative review. Medical-Surgical Nurs J. 2018;7(3).

[18] Kianian T, Lotfi M, Zamanzadeh V, Rezayan A, Hazrati M, Pakpour V (2022). Exploring barriers to the development of home health care in Iran: a qualitative study. Home Health Care Manage Pract.

[19] Rominger R. How to develop and use open-ended surveys/questionnaires. Center for Educational and Instructional Technology Research College of Doctoral studies. University of Phoenix; 2020.

[20] Allen M. The SAGE encyclopedia of communication research methods. SAGE; 2017.

[21] Kyngäs H, Mikkonen K, Kääriäinen M. The application of content analysis in nursing science research. Springer; 2019.

[22] Shufutinsky A (2020). Employing use of self for transparency, rigor, trustworthiness, and credibility in qualitative organizational research methods. OD Practitioner.

[23] Johnson JL, Adkins D, Chauvin S. A review of the quality indicators of rigor in qualitative research. Am J Pharm Educ. 2020;84(1). 10.5688/ajpe7120 PMC7055404 32292186

[24] Shahsavari H, Nasrabadi AN, Almasian M, Heydari H, Hazini A (2018). Exploration of the administrative aspects of the delivery of home health care services: a qualitative study. Asia Pac Family Med.

[25] Moradian ST, Nourozi K, Ebadi A, Khankeh HR (2017). Barriers against providing home health care delivery to ventilator-dependent patients: a qualitative content analysis. Trauma Monthly.

[26] Kiersey R, Coleman A. Approaches to the regulation and financing of home care services in four European countries. Dublin: Health Research Board. 2017;112.

[27] Doshmangir L, Rashidian A, Kouhi F, Gordeev VS (2020). Setting health care services tariffs in Iran: half a century quest for a window of opportunity. Int J Equity Health.

[28] Adamakidou T, Kalokerinou-Anagnostopoulou A (2017). Home health nursing care services in Greece during an economic crisis. Int Nurs Rev.

[29] Megaritis C, Sakellari E, Psychogiou M, Tzenalis A, Krepia V, Charalambous G, et al. editors. Exploring home care nurses’ perceptions regarding their services in economic crisis: a qualitative approach. Nursing forum. Wiley Online Library; 2018. 10.1111/nuf.12281 29968259

[30] Fatemi NL, Moonaghi HK, Heydari A (2018). Exploration of nurses’ perception about professionalism in home care nursing in Iran: a qualitative study. Electron Physician.

[31] Grasmo SG, Liaset IF, Redzovic SE (2021). Home care workers’ experiences of work conditions related to their occupational health: a qualitative study. BMC Health Serv Res.

[32] Arab M, Shahriari M, Keshavarzian A, Abbaszadeh A, Keshvari M (2022). Nurses’ experiences of the ethical values of home care nursing: a qualitative study. Int J Nurs Sci.

[33] Rosenfeld AS (2021). Consider the caregivers: reimagining labor and immigration law to benefit home care workers and their clients. BCL Rev.

[34] Shin JH, Shin I-S, Lee JY (2020). Factors influencing nursing home accreditation-quality ratings in Korea. J Korean Gerontological Nurs.

[35] Desveaux L, Mitchell J, Shaw J, Ivers N (2017). Understanding the impact of accreditation on quality in healthcare: a grounded theory approach. Int J Qual Health Care.

[36] Cristofori V, Brunelli L, Battistella C, Agnoletto A, Catelani A, De Sarno C (2020). How to scale up quality and safety program into home care: an accreditation tool proposal. Eur J Pub Health.

[37] Tang X, Chen X, Wu B, Ma C, Ge S, Sun H (2021). Where are we and what shall we do next? A qualitative study of the quality of home care in Shanghai, China. J Transcult Nurs.

[38] Ajami Sima CK (2018). Information and communication technology in nursing home care services. Health Manage Q.

[39] Jones CD, Jones J, Richard A, Bowles K, Lahoff D, Boxer RS (2017). Connecting the dots: a qualitative study of home health nurse perspectives on coordinating care for recently discharged patients. J Gen Intern Med.

